# Long-Term Survival in Locally Advanced* KRAS* Wild-Type Pancreatic Adenocarcinoma

**DOI:** 10.1155/2019/8598635

**Published:** 2019-07-10

**Authors:** Marion Alhenc-Gelas, Romain Cohen, Pascale Cervera, Jean-Christophe Vaillant, Thierry André

**Affiliations:** ^1^Sorbonne Université, Department of Medical Oncology, AP-HP, Hôpital Saint-Antoine, F-75012 Paris, France; ^2^Sorbonne Université, Department of Pathology, AP-HP, Hôpital Saint-Antoine, F-75012 Paris, France; ^3^Sorbonne Université, Department of Digestive and Hepatobiliary Surgery, AP-HP, Pitié-Salpêtrière Hospital, F-75013 Paris, France

## Abstract

Pancreatic adenocarcinoma remains a cancer associated with a poor prognosis. For locally advanced pancreatic cancer (LAPC), median overall survival is approximately 16 months. Here we report the case of a 52-year-old LAPC patient treated with chemotherapy followed by chemoradiotherapy that was associated with a 14-year complete remission. A peritoneal relapse was then observed and chemotherapy was undergone until the patient died of infectious complications, 17 years after his diagnosis. The tumor was found* KRAS*,* TP53*,* BRCA1, *and* BRCA2 *wild-type. This* KRAS* wild-type LAPC-long survivor case report emphasizes the need to develop molecular approaches to predict LAPC patients' prognosis.

## 1. Introduction

Pancreatic adenocarcinoma (PC) is the fifth leading cause of cancer death and the second leading digestive cancer in incidence in Western countries, with a 5-year survival rate of less than 5% [1, 2]. For locally advanced PC (LAPC), median overall survival (OS) is approximately 16 months [3]. Here we report the case of long survivor of* KRAS *wild-type LAPC who benefited from a therapeutic strategy combining cytotoxic agents and radiotherapy.

## 2. Case Report

On July 2000, a 52-year-old man presented a loss of weight associated with dyspepsia. Ultrasonography showed a 4 cm mass in the head of the pancreas infiltrating biliary ducts, portal vein and an involvement of superior mesenteric artery greater than 180°. No metastatic dissemination was found. Pathological examination of the tumor revealed a moderately differentiated tubular pancreatic adenocarcinoma showing eosinophilic abundant cytoplasm and vesicular nucleus with a clear nucleolus. The patient previously received 10 cycles of a regimen combining gemcitabine 1,000 mg/m(2) as a 10-mg/m^2^/min infusion on day 1 and oxaliplatin 100 mg/m(2) as a 2-hour infusion on day 2 every 2 weeks [4]. Tumor stability was observed while CA19.9 level decreased from 147 U/mL to 47 U/mL. In March 2001, chemoradiotherapy was performed (45 Gy with a 10 Gy boost associated with 5-fluorouracil) and resulted in a radiological tumoral stability, with a normalized CA19.9 level.

Between April 2001 and May 2015, no tumor recurrence was detected. During this period, the patient presented several episodes of hematemesis, attributed to severe ulcerated bulbitis. The bulbitis was likely a side-effect of radiotherapy.

In May 2015, a disease recurrence was observed with jaundice due to an extrinsic obstruction of the common bill duct. Endoscopic biliary catheterism failed and eventually a surgical biliojejunal derivation was performed; peritoneal involvement was observed during the procedure. Chemotherapy was initiated in September 2015, with 5FU and oxaliplatin (FOLFOX regimen). Oxaliplatin was stopped after 7 cycles because of neuropathy and irinotecan was introduced after normalization of bilirubin for 5 more cycles until January 2016, followed with maintenance treatment by capecitabine until June 2016. At this time, disease remained stable with peritoneal diffuse infiltration and biliary duct dilatation.

Then, the patient benefited from a 12-month therapeutic-free interval. A diffuse lymph node tumor progression was detected in July 2017. A combination of capecitabine and oxaliplatin was initiated. Confirmed stable disease was observed and the treatment was withheld in December 2017 because of infectious complications. From January to March 2018, the patient presented several episodes of angiocholitis related to an anastomotic hepaticojejunal stenosis. A percutaneous biliary derivation was successfully performed with adapted antibiotherapy. Nevertheless the patient presented a new septic episode which revealed the appearance of multiples intrahepatic abscesses. CT-scans conducted at this period did not reveal any tumoral progression, but a neoplastic cholangitis could not be excluded from the causes of the biliary obstruction. Unfortunately in March 2018 the patient finally died of this medically challenging infectious complication.

An exploratory molecular analysis was performed on patient's tumor sample. No mutation was detected in* PIK3CA, BRAF, BRCA1, *and* BRCA 2 *genes, neither in* KRAS *and* TP53 *genes. No loss of MMR protein expression was observed (i.e., mismatch repair proficiency).

## 3. Discussion

Here we report the case of a very long survivor of LAPC with* KRAS *wild-type mutational status. Long-term PC survivors are poorly characterized, with few described prognostic clinical, biological, or molecular parameters [5].

The patient was treated with chemoradiotherapy after induction chemotherapy. This strategy has not been demonstrated to improve neither OS (15.2* versus *16.5 months, hazard ratio (HR) 1.03, p=0.83) nor progression-free survival (HR 0.78, p=0.06) in the LAP07 phase III study but it enhances median delay to treatment reintroduction (6.1* versus *3.7 months, 95% confidence interval (95%CI) 4.8 - 7.0 months, p=0.02) [3]. Based on this trial, a prognostic nomogram for LAPC patients with 5 independent prognostic factors of OS has been designed: age, pain, tumor size, albumin, and CA19-9. The PROLAP score dichotomizes LAPC patients into three prognosis groups with median OS of 15.4, 11.7, and 8.5 months (p<0.0001) [6]. According to the nomogram, the predicted median survival time of our 17-year LAPC survivor would have been about 14 months (95%CI 11.75-15.5) and the 4-year survival probability about 4% (95%CI 2-9). Nevertheless LAP07 study brought out the existence of long survivors after chemoradiotherapy who experience long remission periods without antitumor treatments.

Several genetic features have also been identified as prognosis biomarkers of PC patients. In 2012, an association was suggested between the number of mutated oncogenes and survival [7]. Focusing on the 4 main oncogenic drivers in PC (*KRAS, CDKN2A, SMAD4, *and* TP53*), a statistically significant association was found between the number of altered genes (1 or 2* versus *3 or 4) and OS (p=0.041) in a cohort of 79 patients. It is worth noting that these genetic alterations are harbored by the vast majority of PC (> 90% for* KRAS *and* CDKN2A, *75% for* TP53 *and 55% for* SMAD4)* [8, 9]. Nevertheless the prognostic impact of* KRAS *mutation remains a matter of debate. Recently, Bournet and colleagues did not find* KRAS *mutational status as an OS prognostic factor, even if* KRAS *codon 12 mutation was significantly associated with a worse prognosis [10]. Similarly,* KRAS *mutation was not associated with OS in another cohort of 178 PC patients [11].

Interestingly* KRAS *wild-type status might be a predictive factor of response to EGFR inhibitors. In a phase IIb randomized trial evaluating first-line gemcitabine with or without nimotuzumab (a humanized monoclonal antibody acting like an* EGFR *inhibitor), 12-month OS rate was 53.8% versus 15.8% in the* KRAS *wild-type population (HR 0.32, 95%CI 0.13-0.84, p=0.026). It is worth noting that the proportion of* KRAS *wild-type patients in this study was surprisingly much higher than that usually described in literature (26.5% in the combination arm and 41.7% in the gemcitabine arm) [12]. This result suggests that* EGFR *inhibitors might be efficient in first-line treatment for* KRAS *wild-type advanced PC patients.

## 4. Conclusion

Our case report emphasizes the need to develop molecular approaches in the field of PC to predict the patients' prognosis with more accuracy and to promote personalized therapeutic strategies.
